# *Viscum album* neutralizes tumor-induced immunosuppression in a human *in vitro* cell model

**DOI:** 10.1371/journal.pone.0181553

**Published:** 2017-07-18

**Authors:** Carmen Steinborn, Amy Marisa Klemd, Ann-Sophie Sanchez-Campillo, Sophie Rieger, Marieke Scheffen, Barbara Sauer, Manuel Garcia-Käufer, Konrad Urech, Marie Follo, Annekathrin Ücker, Gunver Sophia Kienle, Roman Huber, Carsten Gründemann

**Affiliations:** 1 Center for Complementary Medicine, Institute for Infection Prevention and Hospital Epidemiology, Faculty of Medicine, University of Freiburg, Freiburg, Germany; 2 Verein für Krebsforschung, Arlesheim, Switzerland; 3 Lighthouse Core Facility, Department of Medicine I, Medical Center—University of Freiburg, Faculty of Medicine, University of Freiburg, Freiburg, Germany; Institut National de la Santeet de la Recherche Medicale (INSERM), FRANCE

## Abstract

Tumor cells have the capacity to secrete immunosuppressive substances in order to diminish dendritic cell (DC) activity and thereby escape from immune responses. The impact of mistletoe (*Viscum album*) extracts (VAE), which are frequently used as an additive anti-cancer therapy to stimulate the immune response, is still unknown. Using a human cellular system, the impact of two different VAE (VAEA + VAEI) on the maturation of human dendritic cells and on T cell function has been investigated using flow cytometry, automated fluorescence microscopy and cytokine bead array assays. Furthermore, we examined whether VAEI was able to counteract tumor-induced immunosuppression within this cellular system using a renal cancer cell model. The role of mistletoe lectin (ML) was analyzed using ML-specific antibodies and ML-depleted VAEI. VAEI and VAEA augmented the maturation of dendritic cells. VAEI abrogated tumor-induced immunosuppression of dendritic cells and both processes were partially mediated by ML since ML-depleted VAEI and ML-specific antibodies almost neutralized the rehabilitative effects of VAEI on DC maturation. Using these settings, co-culture experiments with purified CD4^+^ T cells had no influence on T cell proliferation and activation but did have an impact on IFN-γ secretion. The study provides a potential mode-of-action of VAE as an additive cancer therapy based on immunomodulatory effects. However, the impact on the *in vivo* situation has to be evaluated in further studies.

## Introduction

Tumor cells naturally occur in the human organism but usually these cells are detected and eliminated by the immune system, especially by the interaction of antigen presenting cells (APC) and related effector cells, like NK cells or T cells [1,2]. However, tumor cells have the capacity to secrete immunosuppressive substances, like vascular endothelial growth factor (VEGF), prostaglandin E2 (PGE2), and various cytokines [3–6] thus creating an immunosuppressive environment which facilitates an escape from the immune response. Activated dendritic cells (DC) are the most potent APC of the human immune system [7] and are crucial for the specific activation or induction of tolerance of T-lymphocytes [8,9]. In particular, DC have the capacity to cross-present tumor antigens to T cells and thereby activate these cells. Activated cytotoxic CD8^+^ T cells are then able to eliminate tumor cells [10]. Therapeutic strategies to counteract the immunosuppressive environment have recently been successful by e.g. blocking immune checkpoints like CTLA4 or PD1 on T cells with specific antibodies and thereby restoring the cytotoxic capacity of these cells [11,12].

Extracts from the European mistletoe (*Viscum album* L.) are used for the treatment of cancer within concepts of complementary medicine, especially anthroposophical medicine. Some randomized, controlled studies have found prolonged survival and benefits with regarding to quality of life [13,14]. A recent randomized controlled trial found a significant prolongation of overall survival in patients with advanced pancreatic cancer after subcutaneous injections of *Viscum album* extracts (VAE) [15]. Amongst other substances, VAE contains mistletoe lectins (ML), glycoproteins with a cytotoxic A-chain, which have RNA-N-glycosidase activity, and a B chain, which binds to sugar binding sites of the cell surface and allows the A-chain to enter the cell by receptor mediated endocytosis [16,17]. ML are cytotoxic to cancer cells in cell culture in the lower nanogram range [18,19]. However, because ML B-chains also bind to white blood cells and many other cell types, clinically relevant anticancer effects mediated by cytotoxic effects of ML are only seen after direct intra-tumoral application of these preparations [20–22]. On the other hand, ML are potent immune modulators and it has been shown that they induce high numbers of T-cells and eosinophils in the peripheral blood [23,24]. Stimulation of DC maturation has also been shown *in vitro* [25–27]. However, nothing is known about the effects of VAE on the tumor-induced immunosuppression of dendritic cells and the role of ML in this process. In the present study the importance of ML in VAE for the recovery of DC maturation after treatment with tumor-supernatant could be shown.

## Materials and methods

### Ethics statement

Written informed consent was obtained from patients prior to blood donation for research purposes. All experiments conducted on human material were approved by the Ethics committee of the University Freiburg (482/11).

### Mistletoe preparations and mistletoe lectin antibody

Two commercial mistletoe preparations, Iscador® Qu spez (VAEI; Iscador AG, Arlesheim, Switzerland) and abnobaViscum fraxini (VAEA; ABNOBA Heilmittel GmbH, Pforzheim, Germany) were used for the experiments. Mistletoe lectin was isolated and depleted from VAEI by affinity chromatography with immobilized α1-acid glycoprotein. α1-acid glycoprotein was used because of its high and non-selective affinity for all three isoforms of ML [28]. 50 mg of α1-acid glycoprotein (orosomucoid from Sigma-Aldrich, Buchs, Switzerland) was coupled to 2 ml Affi-gel 15 (Bio-Rad, Cressier, Switzerland) according to the manufacturer’s instructions. 200 ml of VAEI (20 mg/ml) was passed through 1 ml of the orosomucoid-coupled gel. This passage was carried out at 0°C to profit by the highly increased affinity of ML to the glycoprotein at cold temperatures [29]. By this procedure 85.6% of the lectins were eliminated from the VAEI. The final solution was sterilized by filtration (0.2 μm pore size). ML concentrations as measured by enzyme-linked immunosorbent assay (ELISA) [30] were 1.28 μg/ml in 20 mg VAEI and 0.18 μg/ml in 20 mg ML-depleted VAEI. According to manufacturer’s data ML concentration of VAEA was 0.89 μg/ml.

### Selection of human peripheral lymphocytes and purified CD4^+^ T cells

Human peripheral blood mononuclear cells (PBMC) were isolated from the blood of healthy adult donors obtained from the Blood Transfusion Centre (University Medical Center, Freiburg, Germany). Venous blood was centrifuged on a LymphoPrepTM gradient (density: 1.077 g/cm3, 20 min, 500 x g, 20°C; Progen, Heidelberg, Germany). Cells were washed twice with PBS (Life Technologies, Darmstadt, Germany), and cell viability as well as cell concentration were determined using the trypan blue exclusion test. Purified CD4^+^ T cells were obtained by CD4^+^ positive selection using the magnetic cell separation method. A cell suspension of 10^8^/mL was prepared and 100 μL of the EasySep® Positive Selection Cocktail was added following an incubation time of 10 minutes at room temperature. Afterwards, 50 μL/mL of the magnetic nanoparticles was added, mixed and incubated at room temperature. After 5 minutes, the cell suspension was adjusted to a total volume of 2.5 mL using recommended medium. The cells were fixed in the EasySep® magnet for 5 minutes and afterwards the supernatant was discarded and the cell number was determined (all products are from StemCell Technologies, Grenoble, France). Cells were cultured in RPMI 1640 full medium (supplemented with 10% heat-inactivated fetal calf serum (GE Healthcare, Freiburg, Germany), 2 mM L-glutamine, 100 U/mL penicillin and 100 U/mL streptomycin (all from Life Technologies, Darmstadt, Germany)) at 37°C in a humidified incubator.

### Generation and maturation of immature monocyte-derived DC

Immature DC were generated after CD14^+^ positive selection. A cell suspension of 10^8^/mL PBMC was prepared, and 100 μL of the EasySep® CD14^+^ Positive Selection Cocktail was added following an incubation time of 10 minutes at room temperature. Afterwards, 50 μL/mL of the magnetic nanoparticles was added, mixed well and incubated at room temperature. After 5 minutes, the cell suspension was adjusted to a total volume of 2.5 mL using the recommended medium. The cells were fixed in the EasySep® magnet for 5 minutes. Afterwards the supernatant was discarded and the cell number determined (all products from StemCell Technologies, Grenoble, France). For generation of DC, the cells were cultured in serum-free CellGro DC Medium (CellGenix, Freiburg, Germany) supplemented with 800 U/mL recombinant human IL-4 (PeproTech, Hamburg, Germany) and 1000 U/mL recombinant human GM-CSF (Leukine sargramostim; Bayer, Leverkusen, Germany). DC were cultivated at a density of 1.6 x 10^6^ cells/mL either alone or supplemented with non-toxic concentrations of *Viscum album* preparations (VAEI, Iscador® Qu Spez 0.5 μg/ml; VAEA, abnobaVISCUM®Fraxini; 0.06 μg/ml), ML-depleted VAEI (0.66 μg/ml, Iscador®) or anti-ML antibody (2.5 μg/ml) or, in another approach, with additional supplementation of 10% tumor supernatant or RPMI 1640 full medium for 3 days at 37°C in a 5% CO_2_/ 95% air atmosphere. DC (DC Stim) were stimulated after 24 hrs using a maturation cocktail (500 ng/mL LPS (Sigma-Aldrich, Taufkirchen, Germany); 50 ng/mL TNF-alpha and 10 ng/mL IL-1beta (both from PeproTech, Hamburg, Germany)).

### Surface receptor analysis and cytokine determination of dendritic cells

The effects of mistletoe preparations and controls on DC maturation were determined by measuring surface receptor expression (anti-human CD83 and CD86 mAbs; both from eBioscience, Frankfurt, Germany; anti-human HLA-DR; BD Biosciences, Heidelberg, Germany) using live cell gating in flow cytometric analysis with a BD FACSCalibur flow cytometer and BD CellQuest Pro. The mediators IL-12p70, IL-6, 8 and TNFα were detected and analyzed in the supernatants of cultured cells using ProcartaPlex human 4-plex assay (Thermo Fisher Scientific, Freiburg, Germany) which was measured with a Luminex® 200™.

### Microscopy and image analysis

The surface marker expression of dendritic cells was analyzed by microscopy after staining with anti-human HLA-DR Alexa 488 and anti-human CD86 Alexa 647 (both from Biolegend, London, UK) and putting the cells in a 96-well black clear bottom plate (BD Bioscience, Heidelberg, Germany). The images were acquired using an Olympus Scan^R High Content Screening Station based on a IX81 stage and using either the 20x 0.45 LUCPLFLN (quantitation) or 40x 0.9 UPLSAPO (close up images) objectives, and Scan^R Acquisition software v. 2.6.2. Analysis of the images was done using Scan^R Analysis software v. 2.6.2. Acquisition and analysis settings were identical for each well. For the analysis, after background subtraction, the segmentation mask used to identify the cells had an intensity threshold for either the Alexa 488 (HLA-DR) and Alexa 647 (CD86) signals, respectively, and used a watershed transform to limit the number of doublets. The Alexa 488 and Alexa 647 intensities, respectively, for all pixels of each cell were summed and defined as the total intensities for each channel, respectively. The means of these total intensity values were then calculated for each well and fluorochrome.

### Co-cultivation of DC and allogeneic purified T cells

For co-cultivation experiments, purified CD4^+^ T cells (as described in selection of human peripheral lymphocytes and purified CD4^+^ T cells) were harvested and washed twice in cold PBS and re-suspended in PBS at a concentration of 5 x 10^6^ cells/mL. For cell division tracking, T cells were incubated for 10 min at 37°C with carboxyfluorescein diacetate succinimidyl ester (CFSE; 5μM: Sigma-Aldrich, Taufkirchen, Germany). The staining reaction was stopped by washing twice with complete RPMI 1640 medium. CFSE^+^ CD4^+^ T cells (5 x 10^5^) were cultured in 96 U-bottomed plates (Greiner, Frickenhausen, Germany) together with 5 x 10^4^ DC that had been incubated in the presence or absence of mistletoe preparations or tumor supernatant for 3 days at 37°C in a 5% CO_2_/ 95% air atmosphere. Mistletoe-treated DCs were further washed extensively. A DC:T cell ratio of 1:10 was used and cells were cultured for 5 days, followed by CD25 surface marker staining (Biolegend, Koblenz, Germany) and flow cytometric analysis. Quantification of IFN-γ in the supernatant was done using ProcartaPlex human IFN-γ simplex assay (Thermo Fisher Scientific, Freiburg, Germany). As controls, CFSE^+^ CD4^+^ T cells were cultured with medium alone (NC) or in the presence of phytohemagglutinin-L (PC; PHA-L; 10 μg/mL; Sigma-Aldrich, Taufkirchen, Germany).

### Preparation of tumor-conditioned supernatants and treatment of cells

The RCC line CLB-TUT was kindly provided from C. Caux (Lyon, France). Cells were cultivated in 75 cm^2^ flasks at a density of 2 x 10^5^ cells/ml in RPMI 1640 full medium (supplemented with 10% heat-inactivated fetal calf serum (GE Healthcare, Freiburg, Germany), 2 mM L-glutamine, 100 U/mL penicillin and 100 U/mL streptomycin (all from Life Technologies, Darmstadt, Germany)). After 48 hrs, supernatants were harvested, filtered, aliquoted and stored at -80°C for further use. As adequate controls medium alone was processed as described above. For experiments tumor-supernatant or medium control was added to DC culture as individually described in the figure legends.

### Data analysis

For statistical analysis, data were processed with Microsoft Excel and SPSS software (IBM, Version 22.0, Armonk, USA). As a preliminary point in statistical analysis, normality of data was confirmed by the Shapiro-Wilk test. Statistical significance was determined by paired two-sample t-tests or by one-way ANOVA followed by Dunnett's post hoc pairwise comparisons. The asterisks represent significant differences from the control group (*P<0.05, **P<0.01, ***P<0.001).

## Results

### *Viscum album* extracts increase the maturation of dendritic cells without affecting cytokine secretion

**Fig 1:**
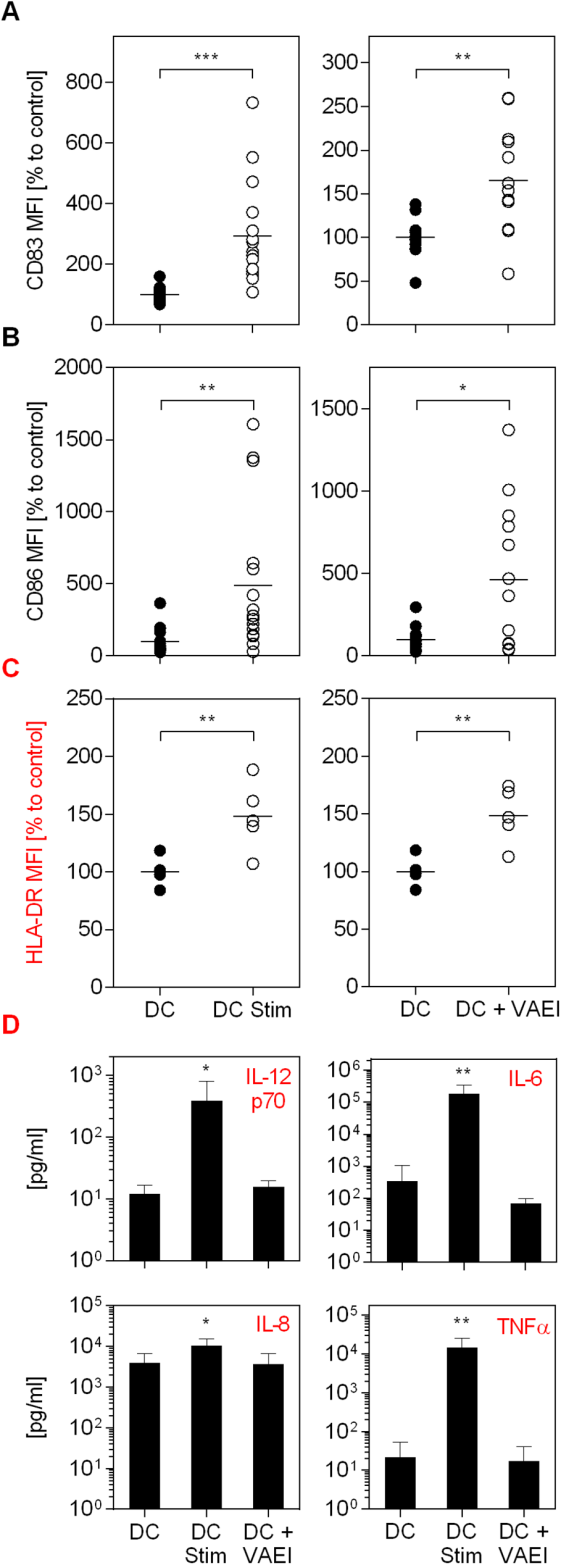
Influence of VAEI on DC maturation and cytokine secretion. CD14^+^ monocytes were matured to immature DC and incubated with medium (DC), maturation cocktail (DC Stim; 500 ng/mL LPS; 50 ng/mL TNF-alpha and 10 ng/mL IL-1beta), or VAEI (Iscador® Qu Spez; 0.5 μg/ml). After cultivation flow cytometric analysis of CD83 **(A)**, CD86 **(B)** and HLA-DR **(C)** expression was carried out. High CD83, CD86 and HLA-DR levels indicate DC maturation. MFI = Mean fluorescence intensity. Data and mean of 16 (DC Stim, CD83 and CD86), 13 (DC + VAEI, CD83 and CD86) or 5 (HLA-DR) individual experiments are presented in relation to untreated cells (DC = 100%). **(D)** The mediators IL-12p70, IL-6, IL-8 and TNFα were detected in the supernatants of cultured cells of 6 independent experiments and analyzed using cytokine bead array assay. Asterisks indicate significant differences between the groups (*P < 0.05, **P < 0.01, ***P < 0.001).

**Fig 2:**
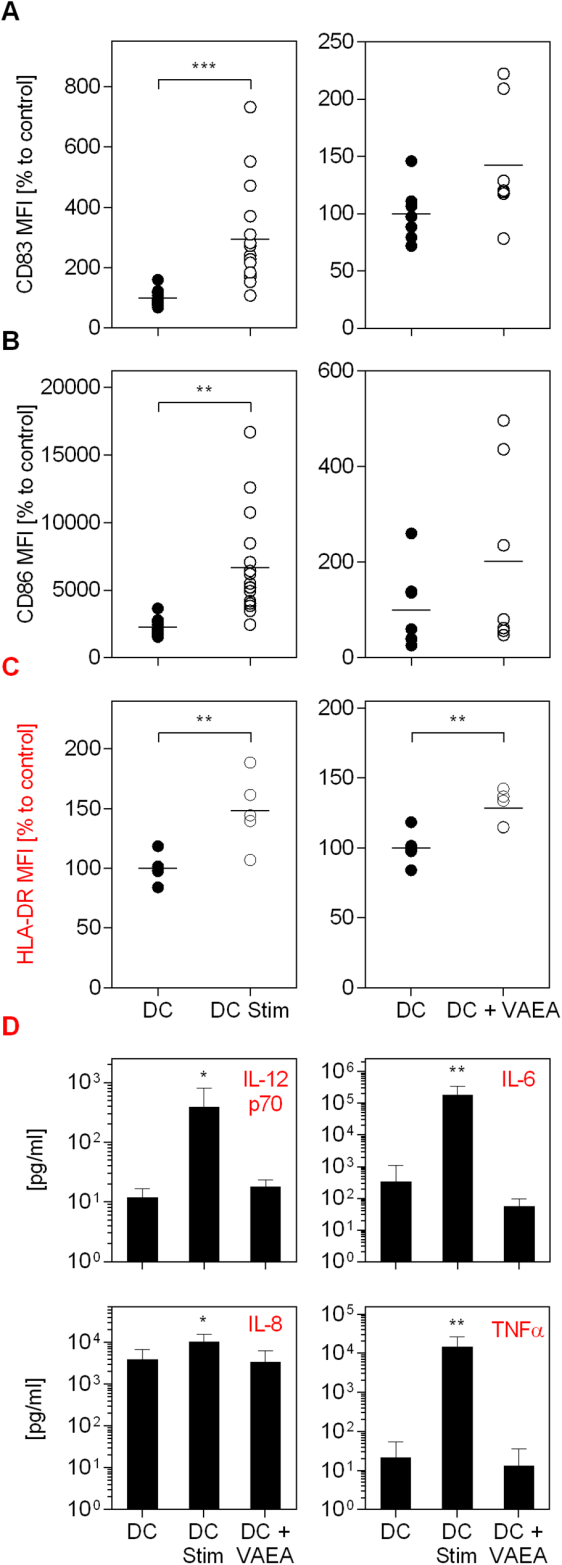
Influence of VAEA on DC maturation and cytokine secretion. CD14^+^ monocytes were matured to immature DC and incubated with medium (DC), maturation cocktail (DC Stim; 500 ng/mL LPS; 50 ng/mL TNF-alpha and 10 ng/mL IL-1beta), or VAEA (DC + VAEA; abnobaVISCUM® Fraxini; 0.06 μg/ml). After cultivation flow cytometric analysis of CD83 **(A),** CD86 **(B)** and HLA-DR **(C)** expression was carried out. High CD83, CD86 and HLA-DR levels indicate DC maturation. MFI = Mean fluorescence intensity. Data and mean of 16 (DC Stim, CD83 and CD86) and 6 (DC + VAEA, CD83 and CD86) or 5 (HLA-DR) individual experiments are presented in relation to untreated cells (DC = 100%). **(D)** The mediators IL-12p70, IL-6, IL-8 and TNFα were detected in the supernatants of cultured cells of 6 independent experiments and analyzed using cytokine bead array assay. Asterisks indicate significant differences between the groups (*P < 0.05, **P < 0.01, ***P < 0.001).

**Fig 3:**
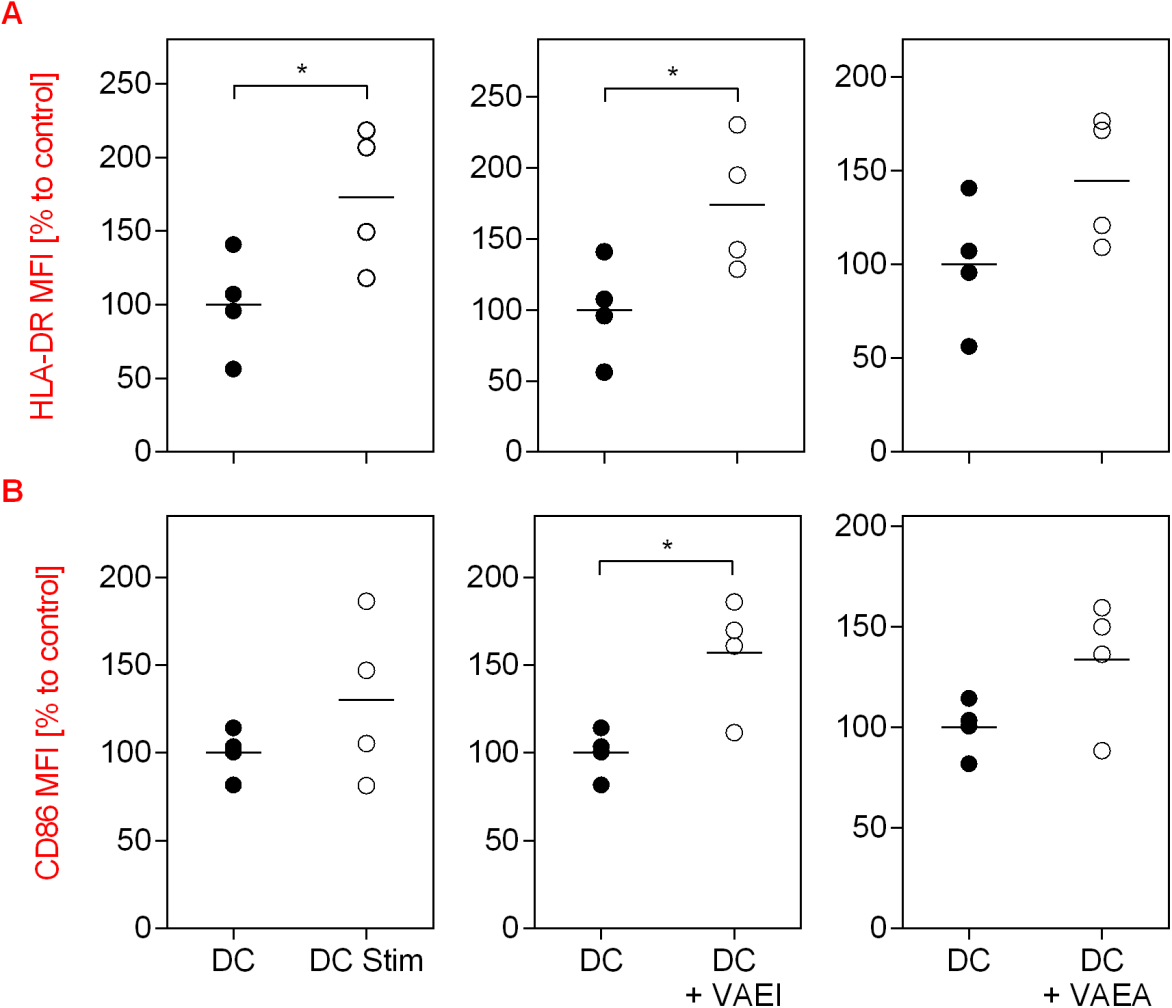
CD86 and HLA-DR expression measured by confocal microscopy. CD14^+^ monocytes were matured to immature DC and incubated with medium (DC), maturation cocktail (DC Stim; 500 ng/mL LPS; 50 ng/mL TNF-alpha and 10 ng/mL IL-1beta), VAEI (DC + VAEI; Iscador® Qu Spez; 0.5 μg/ml) or VAEA (DC + VAEA; abnobaVISCUM® Fraxini; 0.06 μg/ml). After cultivation automated fluorescence microscopy and analysis of HLA-DR **(A)** and CD86 **(B)** expression was carried out. High HLA-DR and CD86 levels indicate DC maturation. MFI = Mean fluorescence intensity. Data and mean of 4 individual experiments are presented in relation to untreated cells (DC = 100%). Asterisks indicate significant differences between the groups (*P < 0.05, **P < 0.01, ***P < 0.001).

The impact of mistletoe preparations on the maturation process of dendritic cells (DC) is shown in Fig 1. As a stimulation control an established maturation cocktail was used. The data demonstrate an increase in the CD83 and CD86 surface marker as well as HLA-DR expression on analyzed cells in the presence of the maturation cocktail (DC Stim) in comparison to immature dendritic cells (DC; Fig 1A, 1B and 1C). CD83, 86 and HLA-DR are markers that are upregulated on mature DC and absent on immature DC. VAEI Iscador® Qu Spez increased the CD83, CD86 and HLA-DR expression to levels comparable to the maturation positive control. This effect was dose- and time-dependent and repetitive administration had no additive benefit on the maturation effects (data not shown). The secretion of DC cytokines IL-12p70, Il-6, IL-8 and TNFα was increased after maturation but not affected by VAEI treatment (Fig 1D). A second VAE preparation (VAEA; abnobaVISCUM®Fraxini) was used to see whether the effects on DC maturation were preparation specific or whether there was a general specificity for *Viscum album* (Fig 2A, 2B and 2C). Analysis of specific surface marker expression demonstrates that VAEA has stimulatory effects on the process of DC maturation, as well. Cytokine levels also remained unchanged upon VAEA treatment of DC (Fig 2D). Effects of VAE on DC maturation were additionally analyzed by automated fluorescence microscopy (Fig 3A and 3B). Expression levels of HLA-DR as well as CD86 were higher on mature compared to immature DC and VAEI-treatment of DC also resulted in an up-regulation of these markers. VAEA had a similar effect on DC maturation. For further experiments VAEI was used.

### *Viscum album* lectins play a major role in mistletoe-induced maturation of dendritic cells

**Fig 4:**
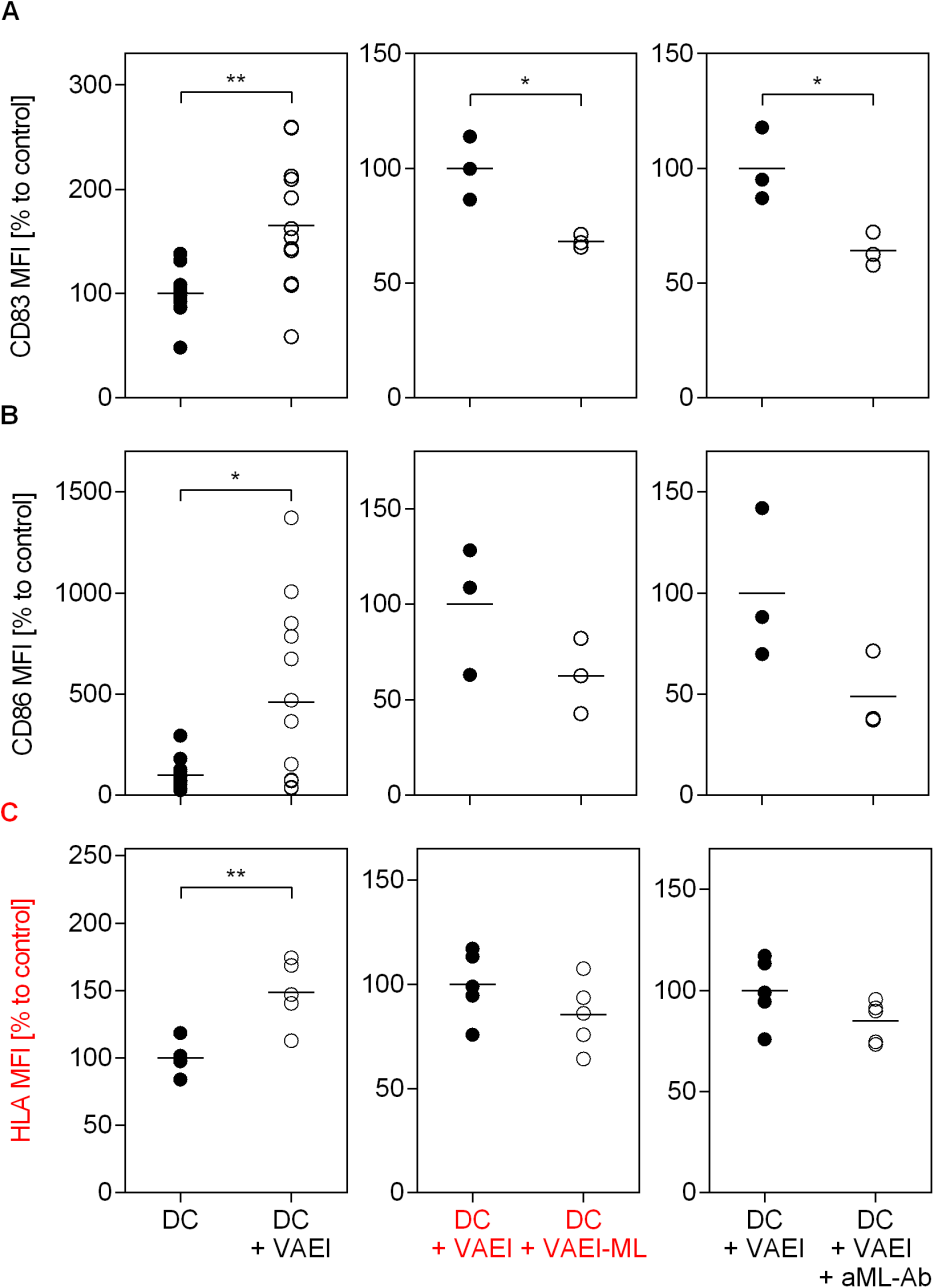
Dependency of VAEI-induced DC maturation on mistletoe lectins. Dendritic cells derived from CD14^+^ monocytes were cultured in medium alone (DC) or supplemented with VAEI (DC + VAEI; Iscador® Qu Spez; 0.5 μg/ml), ML-depleted VAEI (DC + VAEI-ML; Iscador®; 0.66 μg/ml) or VAEI and anti-ML antibody (DC + VAEI + aML-Ab). DC maturation was assessed by flow cytometric analysis of CD83 **(A)**, CD86 **(B)** and HLA-DR **(C)** expression. MFI = Mean fluorescence intensity. Data and mean of 13 (DC + VAEI; CD83 and CD86), 3 (DC + VAEI-ML and DC + VAEI + aML-Ab; CD83 and CD86) or 5 (HLA-DR) individual experiments are represented in relation to untreated cells (DC = 100%) or VAEI-treated DC (DC + VAEI = 100%). Asterisks indicate significant differences between the groups (*P < 0.05, **P < 0.01, ***P < 0.001).

To identify whether mistletoe lectins are involved in the VAE-induced maturation process, DC were treated with either a ML-depleted VAEI (VAEI-ML) preparation or the cells were pre-incubated with an anti-mistletoe lectin (ML) antibody (aML-Ab) (Fig 4). The results assured the influence of the lectin since the VAEI-induced DC maturation, represented by CD83, CD86 and HLA-DR expression, was abrogated when using a ML-depleted VAEI preparation (Fig 4A, 4B and 4C). Furthermore, the presence of a lectin-specific antibody reduced the maturation-inducing effects of VAEI.

### *Viscum album* extract influences DC-mediated T cell stimulation

**Fig 5:**
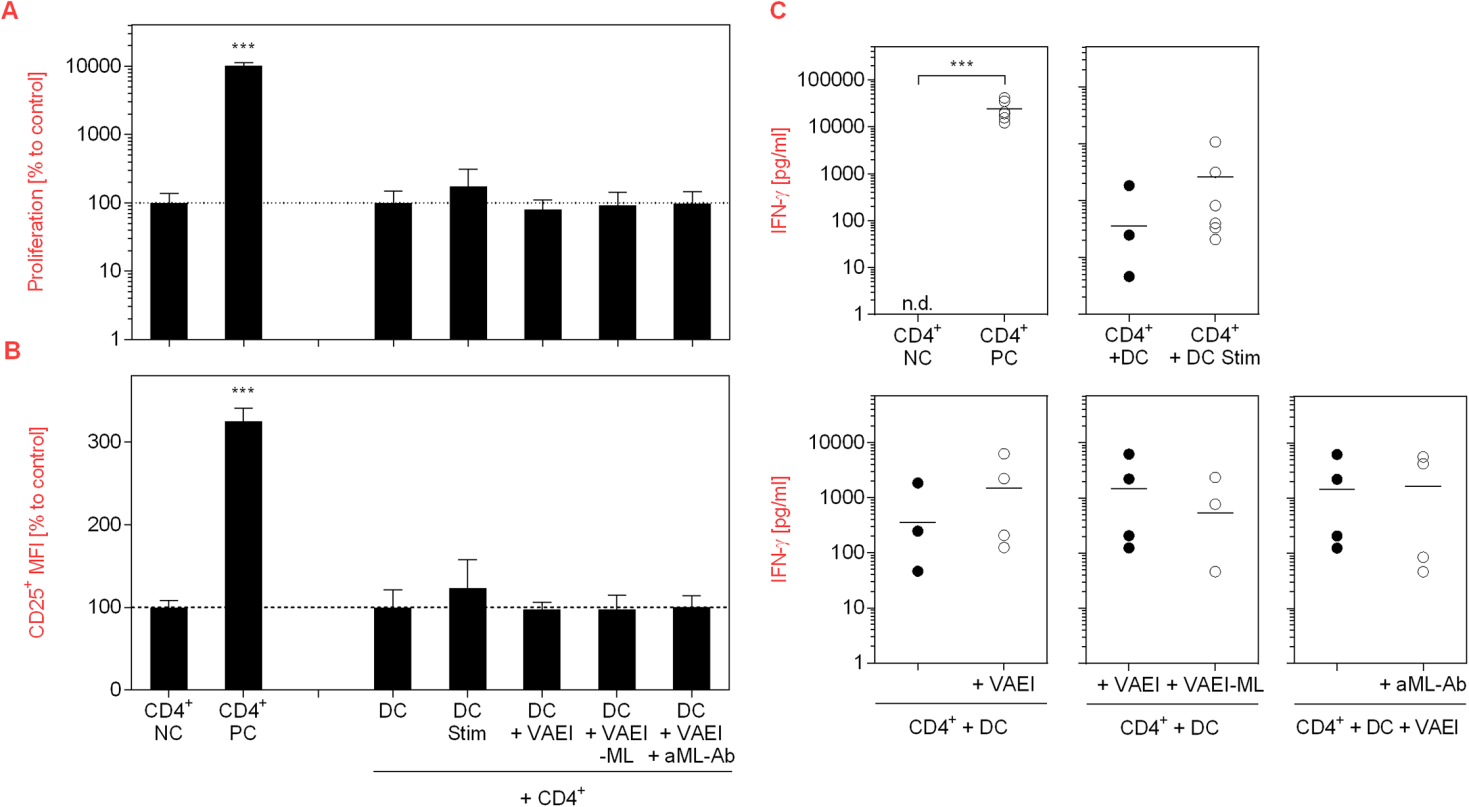
Effects of VAEI-treated DC on T cells. Purified human CD4^+^ T lymphocytes were cultured in the presence of medium (NC) or stimulated with phytohemagglutinin-L (PC; 10 μg/ml). Unstimulated T cells were further co-cultivated with immature DC (DC, CD4^+^), DC treated with a maturation cocktail (DC Stim, CD4^+^; 500 ng/mL LPS; 50 ng/mL TNF-alpha and 10 ng/mL IL-1beta), VAEI-treated DC (DC + VAEI, CD4^+^; Iscador® Qu Spez; 0.5 μg/ml), DC incubated with ML-depleted VAEI (DC + VAEI-ML, CD4^+^; Iscador®; 0.66 μg/ml) or VAEI and anti-ML antibody (DC + VAEI + aML-Ab, CD4^+^; 2.5 μg/ml). **(A)** Cell proliferation analysis was done using CFSE staining and flow cytometry. **(B)** CD25 surface marker expression was analyzed as a second indicator for T cell activation. Data of 6 individual experiments are presented as mean ± SD in relation to untreated T cells (NC) or untreated T cells co-cultured with immature DC (DC, CD4^+^). **(C)** IFN-γ release by T cells was analyzed in the supernatants of the co-cultures of 6 independent experiments using a cytokine bead array assay. Asterisks indicate significant differences between the groups (*P < 0.05, **P < 0.01, ***P < 0.001). Data points with values below the detection limit are not pictured.

Since DC are inducers of T cell activation, the impact of VAEI on this process and the impact of mistletoe lectins was analyzed using a DC-T cell co-culture model (Fig 5). Activation of T cells with PHA (PC) led to a strong increase in their proliferation rate (Fig 5A), CD25 activation marker expression (Fig 5B) and IFN-γ release (Fig 5C). Co-culture of untreated T cells with stimulated DC resulted in a marginal induction of T cell proliferation and CD25 up-regulation, while VAEI-primed DC did not influence the proliferation rate or CD25 expression. Neither did ML-depleted VAEI or VAEI and anti-ML antibody. Furthermore, there was a tendency toward an increase of IFN-γ secretion upon incubation of T cells with stimulated or VAEI-treated DC (Fig 5C).

### *Viscum album* extract abrogates tumor-induced immunosuppression of dendritic cells in a lectin-dependent manner

**Fig 6:**
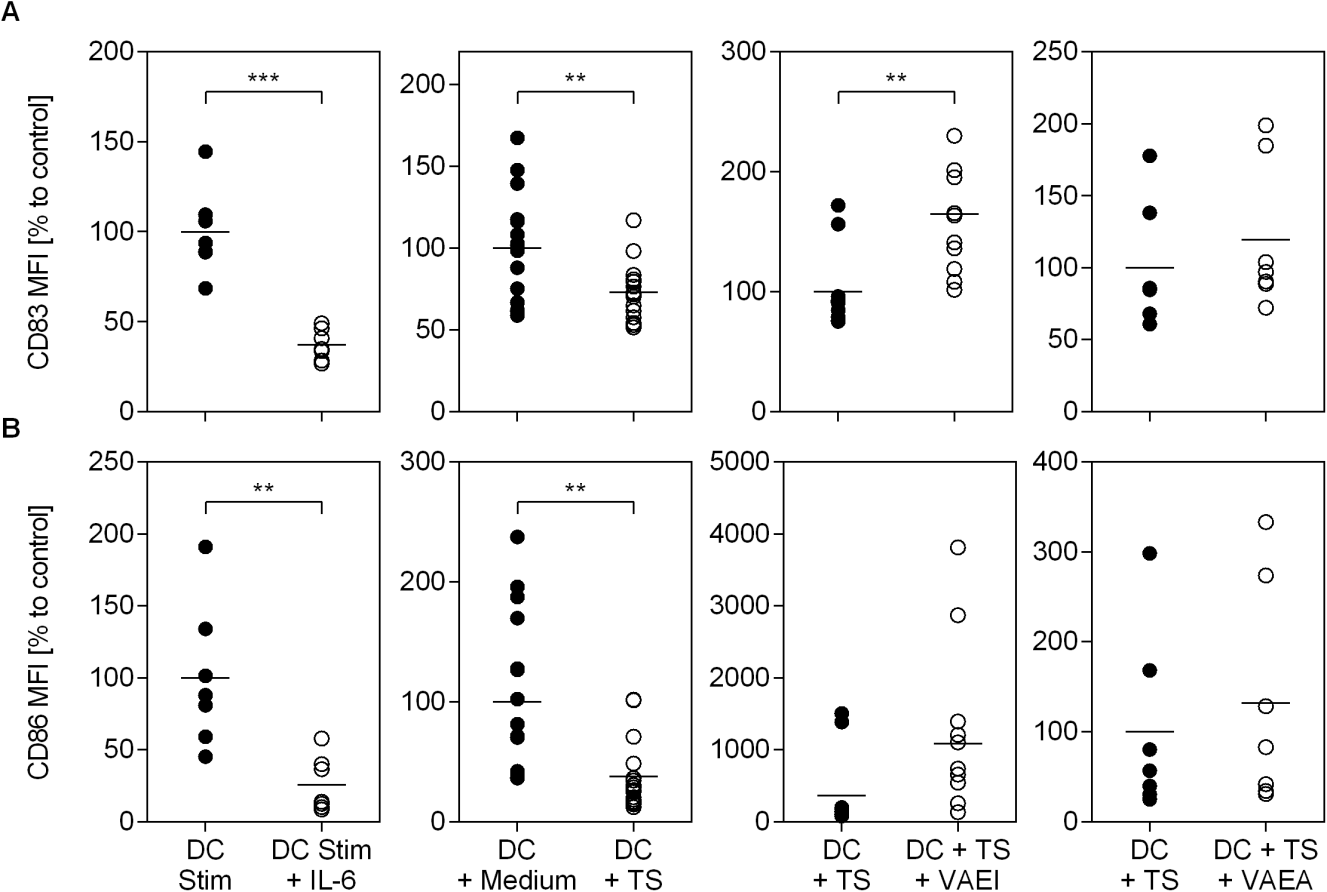
Effects of VAE on tumor-induced suppression of DC maturation. Dendritic cells derived from CD14^+^ monocytes were stimulated with maturation cocktail (DC Stim; 500 ng/mL LPS; 50 ng/mL TNF-alpha and 10 ng/mL IL-1beta) and incubated with IL-6 as an inhibition positive control (DC Stim + IL-6; 12.5 ng/ml). Unstimulated DC were further treated with 10% RPMI 1640 medium (DC + Medium), 10% tumor supernatant of an RCC line (DC + TS) or 10% tumor supernatant and VAEI (DC + TS + VAEI; Iscador® Qu Spez; 0.5 μg/ml) or VAEA (DC + TS + VAEA; abnobaVISCUM® Fraxini; 0.06 μg/ml). DC maturation was assessed by flow cytometric analysis of CD83 **(A)** and CD86 **(B)** surface marker expression. MFI = Mean fluorescence intensity. Data and mean of 7 (IL-6 and DC + TS + VAEA), 16 (DC + TS) or 9 (DC + TS + VAEI) individual experiments are presented in relation to stimulated cells (DC Stim = 100%), stimulated DC cultured in medium alone (DC + Medium = 100%) or stimulated cells cultured with tumor supernatant (DC + TS = 100%). Asterisks indicate significant differences between the groups (*P < 0.05, **P < 0.01, ***P < 0.001).

The established DC *in vitro* model was used to test immunosuppressive effects of tumor-conditioned medium (TS) on the DC maturation process (Fig 6). Interleukin (IL)-6 (12.5 ng/mL) was used as an inhibition positive control as described in the literature. The results demonstrate that IL-6 diminished DC maturation, characterized by a reduction of the surface markers CD83 and CD86 in comparison to stimulated cells alone (Fig 6A and 6B). These results illustrate that the *in vitro* system used has the capacity to depict stimulatory as well as suppressive effects on the maturation process of DC. Moreover, effects of conditioned tumor supernatants of renal cancer cells on DC maturation were analyzed and the data revealed an immunosuppressive influence upon incubation with tumor-conditioned medium. As a control the same amount of RPMI 1640 medium alone was used. Addition of VAE to TS-conditioned DC and analysis of maturation markers was done to see whether VAE has the capacity to reverse immunosuppressive effects. Data illustrate that in the presence of VAEI the tumor-induced immunosuppression was not measurable anymore. Furthermore, abnobaVISCUM®Fraxini (VAEA) was used to examine if this effect could also be measured when using a different VAE preparation. Data show a similar but weaker restorative capacity of VAEA on DC maturation.

**Fig 7:**
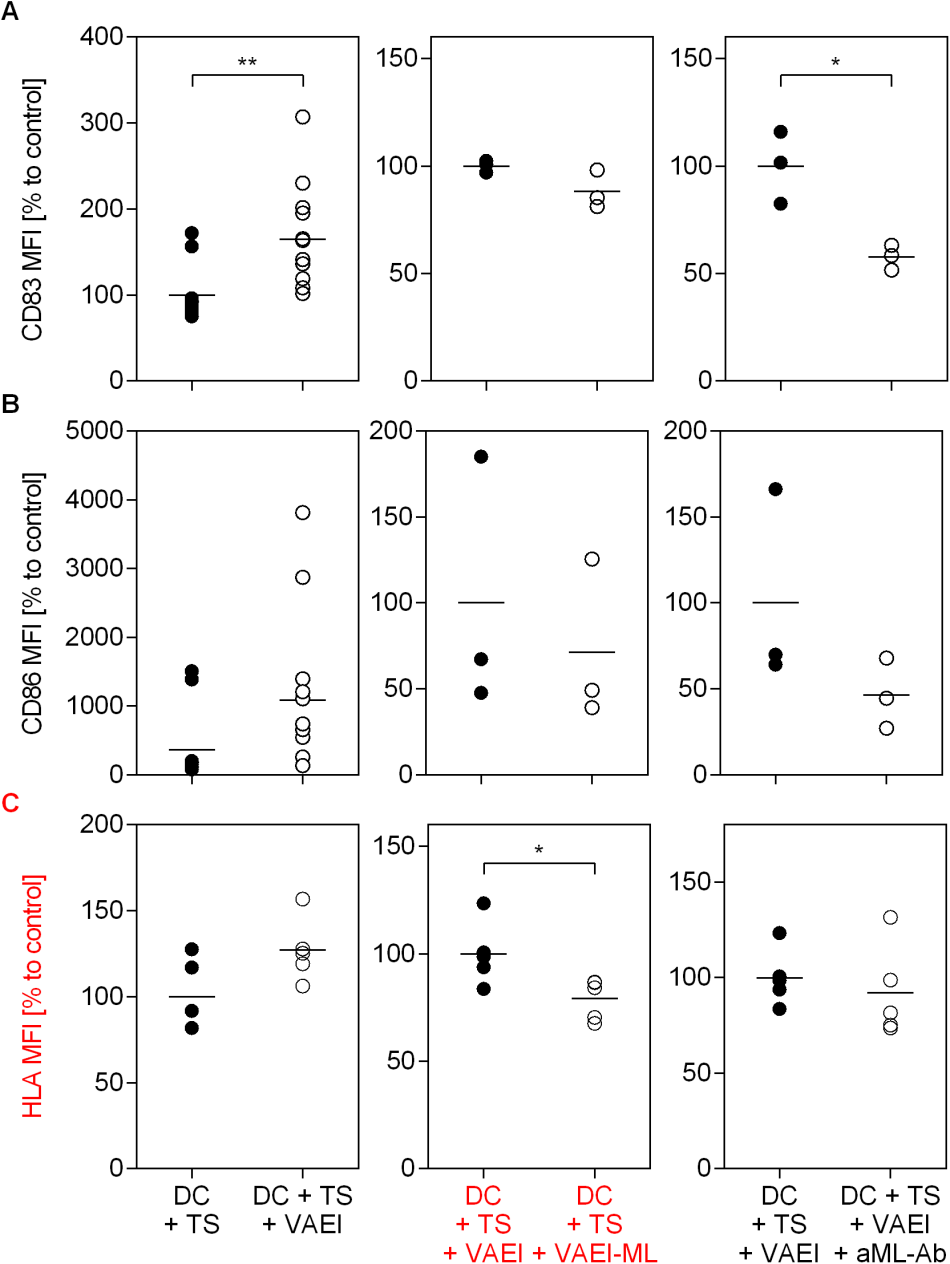
Impact of mistletoe lectins on the restoration of DC maturation with VAEI after tumor-induced immunosuppression. Dendritic cells derived from CD14^+^ monocytes were incubated with 10% tumor supernatant (DC + TS). Further treatment of DC + TS was carried out with VAEI (DC + TS + VAEI; Iscador® Qu Spez; 0.5 μg/ml), ML-depleted VAEI (DC + TS + VAEI-ML; Iscador®; 0.66 μg/ml), or VAEI and anti-ML antibody (DC + TS + VAEI + aML-Ab; 2.5 μg/ml). DC maturation was assessed by flow cytometric analysis of CD83 **(A),** CD86 **(B)** and HLA-DR **(C)** expression. MFI = Mean fluorescence intensity. Data and mean of 12 (DC + TS + VAEI), 3 (DC + TS + VAEI-ML and DC + TS + VAEI + aML-Ab) or 5 (HLA-DR) individual experiments are presented in relation to stimulated and TS-treated cells (DC + TS = 100%), or TS and VAEI-treated DC (DC + TS + VAEI = 100%). Asterisks indicate significant differences between the groups (*P < 0.05, **P < 0.01, ***P < 0.001).

The previous experiments demonstrated that mistletoe lectins are involved in the induction of DC maturation. Therefore, further experiments sought to reveal whether mistletoe lectins are the active compounds of VAE for the restoration of immunosuppression induced by tumor supernatants (Fig 7). For this purpose, DC were either treated with tumor conditioned medium alone or additionally cultured with VAEI, a ML-depleted VAEI preparation or in the presence of anti-ML antibodies (Fig 7A, 7B and 7C). The experiments demonstrate that the restoration of CD83, CD86 as well as HLA-DR expression due to VAEI were abrogated when using a ML-depleted VAEI preparation. The presence of a ML-specific antibody diminished the immunostimulatory effects of VAEI.

**Fig 8:**
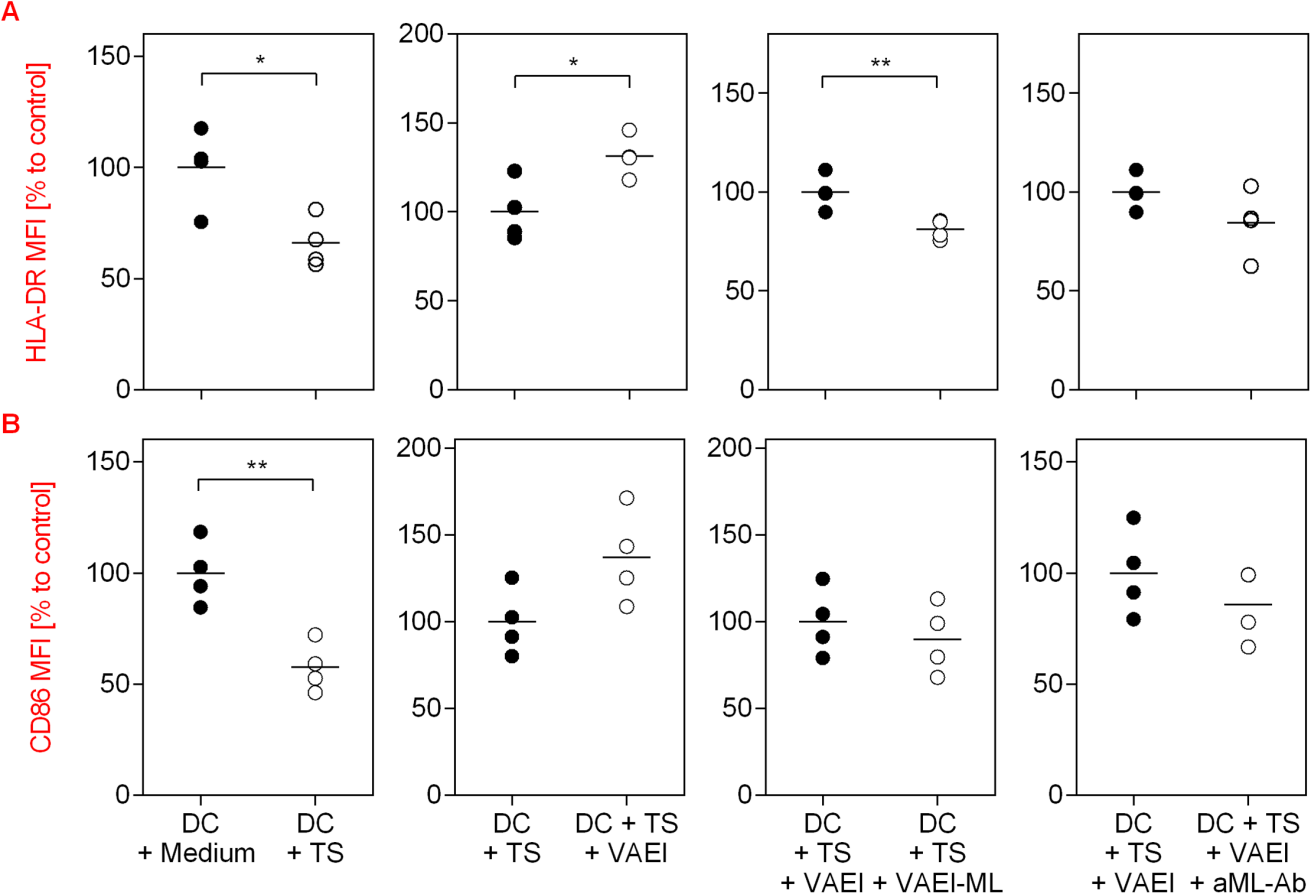
Fluorescence microscopy of the effects of VAEI on the restoration of CD86 and HLA-DR expression on DC after tumor-induced immunosuppression and the impact of mistletoe lectins. Unstimulated CD14^+^-derived DC were treated with 10% RPMI 1640 medium (DC + Medium), 10% tumor supernatant of an RCC line (DC + TS) or 10% tumor supernatant and VAEI (DC + TS + VAEI; Iscador® Qu Spez; 0.5 μg/ml), ML-depleted VAEI (DC + TS + VAEI-ML; Iscador®; 0.66 μg/ml), or VAEI and anti-ML antibody (DC + TS + VAEI + aML-Ab; 2.5 μg/ml). After cultivation automated fluorescence microscopy and quantitative analysis of HLA-DR **(A)** and CD86 **(B)** expression was carried out. MFI = Mean fluorescence intensity. Data and mean of 4 individual experiments are presented in relation to untreated cells (DC = 100%). Asterisks indicate significant differences between the groups (*P < 0.05, **P < 0.01, ***P < 0.001).

The immunosuppressive effects of TS on DC maturation and the lectin-dependent restorative capacity of VAEI on HLA-DR and CD86 expression on DC were additionally supported by automated fluorescence microscopy (Fig 8).

### *Viscum album* enhances DC-mediated IFN-γ release of CD4^+^ T cells after tumor-induced immunosuppression

**Fig 9:**
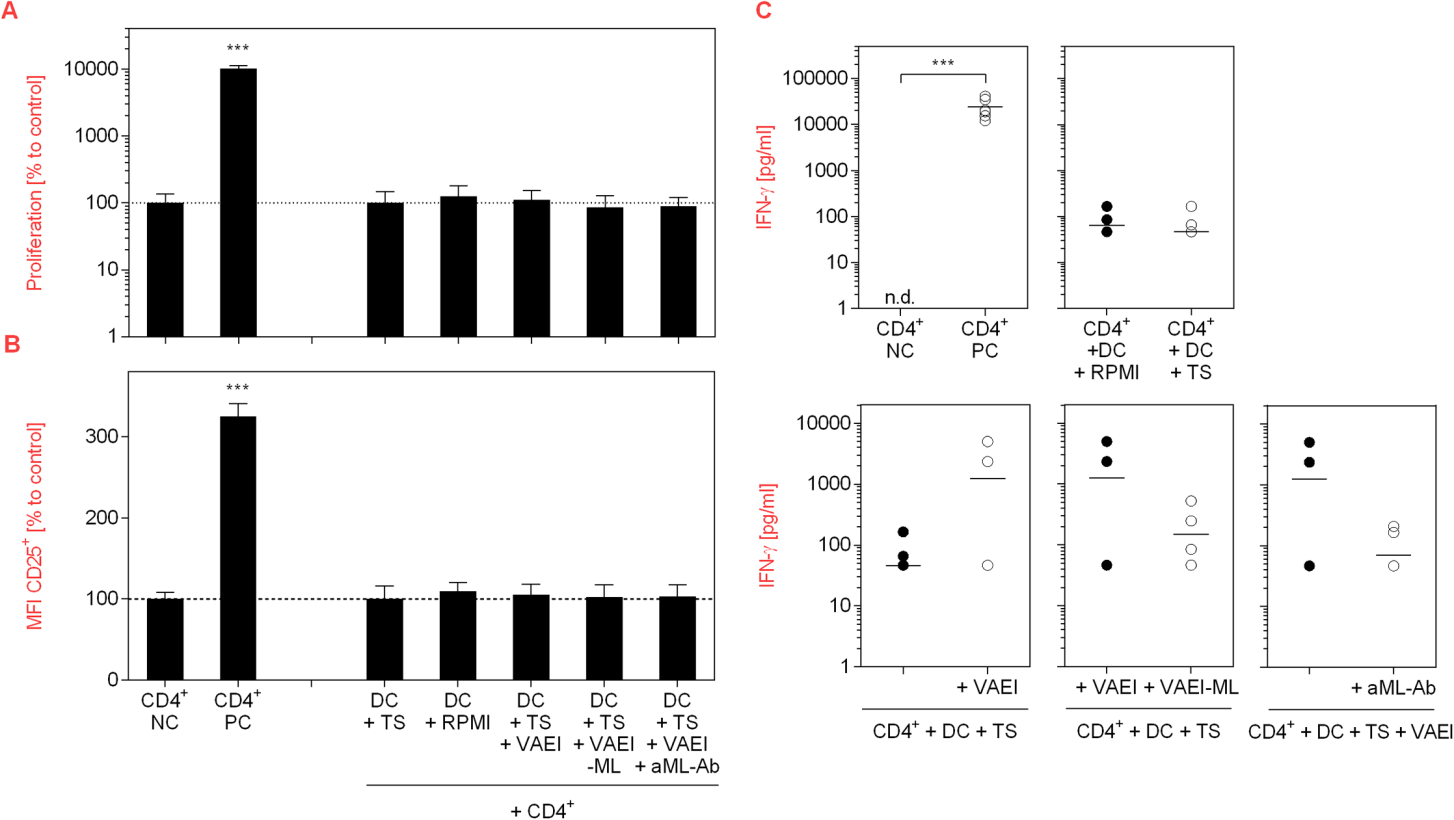
Effects of VAEI-treated DC on T cells after tumor-induced immunosuppression. Purified human CD4^+^ T lymphocytes were cultured in the presence of medium (NC) or stimulated with phytohemagglutinin-L (PC; 10 μg/ml). Co-cultivation of T cells was carried out with unstimulated DC treated with 10% RPMI 1640 medium (CD4^+^ + DC + Medium), 10% tumor supernatant of an RCC line (CD4^+^ + DC + TS^+^), TS and VAEI (CD4^+^ + DC + TS + VAEI; Iscador® Qu Spez; 0.5 μg/ml), TS and ML-depleted VAEI (CD4^+^ + DC + TS + VAEI-ML; Iscador®; 0.66 μg/ml) or TS, VAEI and anti-ML antibody (CD4^+^ + DC + TS + VAEI + aML-Ab, CD4^+^; 2.5 μg/ml). **(A)** Cell division analysis was done using CFSE staining and flow cytometry. **(B)** CD25 surface marker expression was analyzed as a second indicator for T cell activation. Data from 6 individual experiments are presented as mean ± SD in relation to untreated T cells (NC) or T cells co-cultured with TS-treated DC (DC + TS). **(C)** IFN-γ release by T cells was analyzed in the supernatants of 6 independent co-culture experiments using cytokine bead array assay. Asterisks indicate significant differences between the groups (*P < 0.05, **P < 0.01, ***P < 0.001). Data points with values below the detection limit are not pictured.

To investigate if the restorative effect of VAEI on DC maturation after TS treatment has an effect on T cell response, co-culture experiments of TS-treated DC with CD4^+^ T cells were carried out. No differences in T cell proliferation rate or CD25 surface marker expression were measured between CD4^+^ T cells cultured with medium-treated DC and DC treated with TS and VAEI, VAEI-ML or VAEI and anti-ML antibody (Fig 9A and 9B). However, there was a strong trend toward IFN-γ up-regulation by CD4^+^ T cells upon co-cultivation with TS and VAEI-treated DC compared to TS-treated DC (Fig 9C). Furthermore, reductions in the cytokine levels after co-cultivation with TS and VAEI-ML-treated DC or DC incubated with TS, VAEI and anti-ML antibody revealed an impact of mistletoe lectins.

## Discussion

This study provides a potential mode-of-action of VAE as an immune stimulator and thereby gives evidence for the use of VAE as an additive therapeutic anti-cancer strategy. The results demonstrate that the VAE preparations have a maturation-inducing effect on human dendritic cells, represented by an up-regulation of CD83, CD86 and HLA-DR expression. The fact that effects were somewhat stronger when using Iscador® Qu Spez (VAEI) compared to abnobaVISCUM®Fraxini (VAEA) was most probably due to the use of different non-toxic concentrations (0.5 μg/ml and 0.06 μg/ml), which is most likely related to the different amount of mistletoe lectins in the two preparations [20,23]. Since DC are a key player in the regulation of immune responses and since maturation goes along with enhanced cytokine release [8], effects of VAE on the secretion of DC cytokines, such as IL-12p70, IL-6, IL-8 and TNFα were analyzed. Due to the fact that VAE stimulated the maturation of DC, it can be assumed that this process is associated with a modulation of DC cytokines, as well. The presented data, however, demonstrate that VAE has no influence on DC cytokine secretion and thus stands in contrast to earlier studies [26,27] which showed a dose-dependent increase of IL-6, IL-8 and IL-12. This inconsistency could be explained with the use of higher concentrations of VAE (in these studies 15–20 μg/mL), since the type of mistletoe preparation was identical. Preliminary experiments showed that when using our protocol these concentrations were toxic to the cells. Replacement of the culture medium and additional supplementation of IL-4 and GM-CSF as done by Saha et al. (2016; 27) as well as Elluru et al. (2008; 26) seems to prolong cell survival and might therefore allow treatment of DC with higher VAE concentrations.

Co-culture experiments with purified allogeneic CD4^+^ T cells showed a marginal influence of VAEI-primed DC on T cell proliferation, CD25 expression and IFN-γ secretion. Experiments from Elluru et al. (2008; 26) also demonstrate that mistletoe preparations stimulate the proliferation of allogeneic CD4^+^ T cells and promote IFN-γ release. Again, the use of higher VAE concentrations might explain the stronger effects observed in this study. IFN-γ plays an important role in promoting protective anti-cancer immune responses due to an enhancement of MHC class I and II expression on tumor cells and on DC [31].

Mistletoe lectins (ML) are the major active components of VAE. Their apoptotic effects on malignant cells and their potential as immune modulators have been widely described [23,32–34]. In order to investigate the role of ML in the maturation-inducing effect of VAEI, ML-depleted VAEI preparations as well as anti-ML antibodies have been used. VAEI lacking ML failed to induce DC maturation and incubation of VAEI-treated, immature DC with anti-ML antibodies effectively inhibited DC maturation, as well. These results demonstrate that ML is at least in part responsible for stimulating DC maturation with VAEI in the *in vitro* assay used. In co-culture experiments with CD4^+^ T cells the role of ML was not as clear. Since VAEI failed to induce T cell proliferation or CD25 expression, ML-depleted VAEI or anti-ML antibodies did not have an impact, either. However, there was a tendency toward IFN-γ reduction after treatment of DC with VAEI-ML compared to VAEI-treated DC.

One very effective survival strategy of tumors is the production of an immunosuppressive microenvironment to escape the immune response. Amongst other parameters, DC maturation is also inhibited by the tumor microenvironment [5,6], which is characterized by a reduced expression of CD83 and CD86 [12,35]. In our study incubation of DC with tumor supernatant significantly lowered the expression of CD83, CD86 and HLA-DR, confirming the immunosuppressive effects of the tumor. However, DC maturation was not affected after addition of VAEI to DC treated with tumor supernatant, suggesting that VAEI might be able to at least partly restore the immune function which was down-regulated by the immune-evasion effectors of the tumor. Further experiments confirmed the role of ML in this process. Treatment of DC primed with tumor supernatant with a ML-depleted VAEI preparation or addition of anti-ML antibodies did not result in a recovery of DC maturation, thus showing that ML are also crucial for the restorative effects of VAEI in the chosen *in vitro* model of the immune function. The restorative capacity of VAEI on TS-treated DC was also measurable in co-culture experiments with T cells. CD4^+^ T cells co-cultured with TS-treated DC revealed higher cytokine levels after additional incubation with VAEI. This effect was lectin-dependent, as well.

## Conclusion

The results of this study provide further evidence for a mode-of-action of the anti-cancer effects of mistletoe preparations via stimulation of DC maturation. Furthermore, the results obtained with this *in vitro* assay point to a possible role for mistletoe lectins in counteracting the tumor-induced immunosuppression of DC. Further experiments should clarify whether or not this effect is due to a specific interaction of ML with DC and focus on identifying the molecular mechanisms which are involved.

## Supporting information

S1 Table Datasets underlying Figs 1–9. (XLSX)
