# Correction: *Viscum album* neutralizes tumor-induced immunosuppression in a human *in vitro* cell model

**DOI:** 10.1371/journal.pone.0187786

**Published:** 2017-11-02

**Authors:** The PLOS ONE Staff

There are multiple errors in the Author Contributions. The publisher apologizes for the errors. The correct contributions are:

Conceptualization: Carmen Steinborn, Roman Huber, Carsten Gründemann.

Data curation: Carmen Steinborn, Amy Marisa Klemd, Manuel Garcia-Käufer, Carsten Gründemann.

Formal analysis: Carmen Steinborn, Ann-Sophie Sanchez-Campillo, Sophie Rieger, Marieke Scheffen, Barbara Sauer, Manuel Garcia-Käufer, Konrad Urech, Carsten Gründemann.

Funding acquisition: Roman Huber, Carsten Gründemann.

Investigation: Carmen Steinborn, Amy Marisa Klemd, Ann-Sophie Sanchez-Campillo, Sophie Rieger, Marieke Scheffen, Barbara Sauer, Konrad Urech, Marie Follo, Carsten Gründemann.

Methodology: Barbara Sauer, Carsten Gründemann.

Project administration: Roman Huber, Carsten Gründemann.

Resources: Konrad Urech, Roman Huber, Carsten Gründemann.

Software: Manuel Garcia-Käufer.

Supervision: Roman Huber, Carsten Gründemann.

Validation: Carmen Steinborn, Amy Marisa Klemd, Ann-Sophie Sanchez-Campillo, Sophie Rieger, Marieke Scheffen, Barbara Sauer, Annekathrin Ücker, Gunver Sophia Kienle, Carsten Gründemann.

Visualization: Carmen Steinborn, Carsten Gründemann.

Writing—original draft: Carmen Steinborn, Konrad Urech, Marie Follo, Carsten Gründemann.

Writing—reviewing & editing: Carmen Steinborn, Annekathrin Ücker, Gunver Sophia Kienle, Roman Huber, Carsten Gründemann.
